# Inflammatory mechanisms in brain edema from 1,2-dichloroethane poisoning: a review

**DOI:** 10.3389/fneur.2026.1748770

**Published:** 2026-01-29

**Authors:** Yun Tian, Haibin Liu

**Affiliations:** 1 Department of Ultrasound, Yantaishan Hospital, Yantai, Shandong Province, China; 2 Department of Emergency Medicine, North Campus, Yantaishan Hospital, Yantai, China

**Keywords:** 1,2-dichloroethane, blood–brain barrier (BBB), brain edema, cytokines, neuroinflammation, oxidative stress

## Abstract

1,2-Dichloroethane (1,2-DCE) is a synthetic halogenated hydrocarbon widely used in polyvinyl chloride (PVC) production and as an industrial solvent. Prolonged or high-level exposure to 1,2-DCE can cause severe central nervous system injury, with brain edema being one of its major pathological manifestations. Recent studies have revealed that inflammation plays a pivotal role in the onset and progression of 1,2-DCE-induced brain edema. Activation of microglia and astrocytes triggers the release of proinflammatory cytokines such as TNF-*α*, IL-1β, and IL-6, which in turn disrupt the integrity of the Blood–Brain Barrier (BBB) and increase vascular permeability. Moreover, oxidative stress and mitochondrial dysfunction amplify inflammatory signaling through the Mitogen-Activated Protein Kinase (MAPK)–Nuclear factor kappa-light-chain-enhancer of activated B cells (NF-κB) pathway, promoting endothelial injury and cytotoxic edema. This review summarizes current understanding of the inflammatory mechanisms underlying brain edema following 1,2-DCE intoxication, emphasizing the interplay among oxidative stress, cytokine signaling, and BBB disruption. These findings not only elucidate the molecular basis of 1,2-DCE-induced neurotoxicity but also highlight the clinical relevance of targeting inflammation and oxidative stress for therapeutic intervention. In addition, potential therapeutic strategies targeting inflammatory signaling cascades and oxidative damage are discussed, providing insights into future directions for prevention and treatment research.

## 1 Introduction

1,2-Dichloroethane (1,2-DCE) is a chlorinated hydrocarbon widely used as an industrial solvent and an intermediate in the production of polyvinyl chloride (PVC). Due to its high volatility and lipid solubility, occupational exposure mainly occurs through inhalation or dermal absorption (1). It should be noted that dichloroethane exists as two structural isomers, 1,1-dichloroethane and 1,2-dichloroethane, which exhibit markedly different toxicological properties. This review exclusively focuses on 1,2-DCE due to its well-documented neurotoxicity. In developing countries, such as China, occupational exposure to 1,2-DCE has been frequently reported among workers in industries such as shoemaking, electronics, and printing, where poor ventilation and inadequate protection are common. Several outbreaks of 1,2-DCE-induced toxic encephalopathy have been documented, with brain edema identified as the major cause of neurological symptoms and mortality (1–3).

Globally, 1,2-DCE ranks among the most hazardous volatile organic compounds (VOCs) regulated by environmental and occupational health agencies due to its widespread industrial use and high toxicity. According to the Toxicological Profile for 1,2-Dichloroethane published by the Agency for Toxic Substances and Disease Registry (ATSDR, CDC), 1,2-DCE is classified as a hazardous air pollutant with well-documented neurotoxic effects, and occupational inhalation exposure represents a major route of human intoxication (4). The report further highlights central nervous system injury, including brain edema, as a critical adverse outcome associated with acute and subacute exposure. However, comprehensive mechanistic studies and standardized diagnostic criteria for 1,2-DCE-induced neurotoxicity remain limited, especially in developing regions.

Occupational exposure to 1,2-DCE can lead to acute or subacute toxic encephalopathy, in which the central pathological feature is central nervous system injury characterized predominantly by brain edema. Histopathological studies have revealed extensive cytotoxic and vasogenic edema, neuronal swelling and degeneration, perivascular edema, and marked astrocytic necrosis, particularly involving the subcortical white matter, bilateral globus pallidus, and cerebellar dentate nuclei (3, 5, 6).

Although previous studies have described the neuroimaging and clinical manifestations of 1,2-DCE-induced encephalopathy, the molecular mechanisms underlying brain edema remain poorly understood. Increasing evidence suggests that inflammation, oxidative stress, and BBB disruption may play key roles in its pathogenesis. Despite increasing clinical awareness, the molecular events linking exposure to cellular injury are still poorly characterized, and most studies remain descriptive rather than mechanistic. Clarifying these inflammatory and oxidative pathways is crucial for identifying potential therapeutic targets and improving prognosis in affected individuals.

While 1,2-DCE exerts systemic toxicity affecting multiple organs, including the liver, kidney, and cardiovascular system, central nervous system injury—particularly brain edema—represents the most critical determinant of acute neurological deterioration and mortality. These systemic toxicities are thought to share common upstream mechanisms involving oxidative stress and inflammation (6–11). Given the breadth of systemic toxicity caused by 1,2-DCE, this review focuses specifically on inflammation-centered mechanisms driving brain edema, rather than providing an exhaustive survey of all toxicological outcomes.

Therefore, this review integrates current clinical and experimental findings to elucidate the inflammatory mechanisms underlying 1,2-DCE-induced brain edema, emphasizing the interplay among oxidative stress and BBB disruption. Furthermore, it highlights the potential of targeting key signaling pathways, such as NF-κB, MAPK, and NLRP3 inflammasome, as experimental intervention mapping.

## 2 Pathological manifestations of brain edema in 1,2-DCE poisoning

### 2.1 BBB disruption

The BBB is a highly specialized structure formed by brain microvascular endothelial cells connected through tight and adherens junctions, supported by pericytes, astrocytic end-feet, and the basal lamina, which together maintain the brain’s ionic and metabolic homeostasis (12, 13). Histopathological examinations following 1,2-DCE exposure have consistently revealed extensive BBB disruption, as evidenced by endothelial cell swelling, vacuolar degeneration, and marked perivascular edema. Experimental studies have shown significantly increased brain water content and enhanced vascular permeability in affected animals, particularly within the subcortical white matter, globus pallidus, and cerebellar dentate nucleus—regions commonly involved in toxic encephalopathy (14–17). These structural abnormalities are frequently accompanied by widening of perivascular spaces, loosening of endothelial contacts, and astrocytic swelling. Recent reports further describe the presence of reactive astrocytes and glial morphological alterations in these edematous areas, suggesting a close association between BBB injury and diffuse vasogenic edema (18, 19). In addition to these classical pathological features, recent case reports have revealed that 1,2-DCE–induced toxic encephalopathy may present with atypical neuroimaging manifestations, including symmetrical hyperintensities along the cortico–medullary junction on diffusion-weighted imaging (DWI), mimicking neuronal intranuclear inclusion disease (NIID) (20). These findings further expand the neuroimaging spectrum of 1,2-DCE–related brain edema and underscore the importance of detailed occupational exposure history in the differential diagnosis of unexplained encephalopathy.

Taken together, BBB disruption and oxidative–mitochondrial injury constitute key pathological features of 1,2-DCE–induced brain edema, creating a permissive microenvironment for subsequent inflammatory activation.

In addition to these pathological alterations, structural factors involved in water transport and extracellular matrix integrity further contribute to edema progression. Experimental evidence indicates that 1,2-DCE exposure disrupts aquaporin expression and upregulates matrix metalloproteinases, thereby facilitating water influx, extracellular matrix degradation, and blood–brain barrier destabilization (21).

These pathological and structural changes collectively establish the substrate upon which inflammatory signaling cascades are initiated and amplified, as discussed in the following section.

### 2.2 Oxidative stress and mitochondrial dysfunction

In parallel with BBB injury, 1,2-DCE poisoning induces extensive oxidative stress and mitochondrial damage, which are prominently observed in brain tissue and are closely associated with the development of cytotoxic and vasogenic edema. Histological and ultrastructural examinations have revealed increased production of reactive oxygen species (ROS), widespread lipid peroxidation, and loss of mitochondrial integrity in neurons and glial cells. Affected regions exhibit mitochondrial swelling, disrupted cristae architecture, and condensed or fragmented nuclei, suggestive of oxidative injury-induced apoptosis and energy failure (15, 22, 23). These morphological alterations are especially pronounced in glia-rich areas, indicating a selective vulnerability of astrocytes and oligodendrocytes to redox imbalance. Moreover, accumulation of electron-dense mitochondria and intracellular vacuoles has been observed in the subcortical white matter and cerebellum following subacute exposure (22, 23). These pathological features contribute to intracellular fluid accumulation and increased membrane permeability, thereby facilitating brain edema formation.

Taken together, BBB disruption, oxidative stress, and mitochondrial dysfunction represent key pathological features of brain edema following 1,2-DCE exposure.

Clinical and imaging studies of occupational 1,2-DCE poisoning consistently report diffuse cerebral edema, white matter lesions, and basal ganglia involvement (24–28), supporting the translational relevance of experimental findings and underscoring inflammation-associated neurovascular injury as a shared pathological hallmark.

## 3 Molecular and cellular inflammatory mechanisms involved in 1,2-DCE–induced brain edema

Recent experimental and clinical studies have progressively shifted the understanding of 1,2-DCE–induced neurotoxicity from descriptive pathological observations toward mechanistically defined inflammatory cascades. Within this evolving framework, accumulating evidence indicates that inflammation is not merely a secondary response to tissue injury, but rather serves as a central driver of brain edema initiation and progression following 1,2-DCE exposure.

Rather than operating as isolated pathways, inflammatory processes appear to be hierarchically organized and temporally coordinated, beginning with early metabolic activation and oxidative stress, followed by amplification through canonical signaling pathways, and ultimately culminating in sustained neurovascular dysfunction. Upon exposure, metabolic byproducts such as 2-chloroethanol activate glial and endothelial cells, triggering excessive cytokine release, extracellular matrix degradation, and tight-junction disassembly. In parallel, disruption of neurotransmitter homeostasis has emerged as an additional early neurotoxic event. Experimental studies demonstrate that oxidative stress induced by 1,2-DCE leads to glutamate accumulation and altered GABA metabolism, thereby exacerbating neuronal excitotoxicity and sensitizing the neurovascular unit to subsequent inflammatory injury (29). Consistent with this notion, astrocyte-specific upregulation of CYP2E1 mediates 2-chloroethanol–induced oxidative stress and mitochondrial dysfunction, providing an upstream metabolic basis for inflammatory amplification (23).

In line with these findings, metabolic activation of 1,2-DCE and its toxic intermediates has been shown to trigger oxidative stress–dependent activation of p38 MAPK signaling, which precedes and amplifies downstream inflammatory responses (16, 17). These early events are subsequently followed by MAPK-dependent NF-κB activation as a central inflammatory amplifier, and by secondary mechanisms that sustain and propagate neuroinflammation, including inflammasome activation and glial crosstalk. The following subsections summarize these processes according to their functional roles within the inflammatory cascade. Recent reviews further emphasize that the inflammatory response to 1,2-DCE extends beyond microglial activation and cytokine release, encompassing oxidative stress–driven p38 MAPK/NF-κB signaling, cortical demyelination, and apoptosis, thereby reinforcing inflammation as a key initiator of neurotoxic cascades (6). Compared with prior reviews that primarily catalog pathological features and isolated molecular changes, the present review emphasizes the hierarchical organization and temporal coordination of inflammatory cascades driving brain edema following 1,2-DCE exposure.

### 3.1 p38 MAPK–NF-κB signaling pathway as an early inflammatory amplifier

Activation of the mitogen-activated protein kinase (MAPK) and nuclear factor-κB (NF-κB) signaling pathway represents one of the earliest and most robust inflammatory responses following 1,2-DCE exposure. In mice, Jin et al. found that subacute 1,2-DCE exposure activated the p38 MAPK–NF-κB inflammatory axis, leading to marked upregulation of IL-1β, TNF-*α*, ICAM-1, VCAM-1, and iNOS, accompanied by astrocyte and microglia activation (GFAP and Iba-1 expression) (16, 17). These mediators directly compromise endothelial tight-junction integrity and amplify blood–brain barrier (BBB) permeability, positioning MAPK–NF-κB signaling as a central early amplifier of inflammatory injury. Pharmacological inhibition of p38 MAPK, NF-κB, MMP-9, or IL-1β significantly reduced MMP-9 expression, preserved tight-junction integrity, and alleviated brain edema. These findings demonstrate that MMP-9–dependent tight-junction degradation, regulated by the p38 MAPK–NF-κB cascade, is a major mechanism underlying BBB disruption in 1,2-DCE intoxication (16, 17). Moreover, 1,2-DCE-induced cortical demyelination has been linked to astrocytic AQP4 downregulation and oligodendrocytic MBP loss, possibly mediated via MAPK signaling, further exacerbating the inflammatory injury to BBB and white matter (30).

### 3.2 NLRP3 inflammasome activation as a secondary inflammatory escalation mechanism

Downstream of initial cytokine induction, persistent oxidative stress and mitochondrial dysfunction promote activation of the NLRP3 inflammasome, representing a secondary escalation step in the inflammatory cascade. NLRP3 activation drives caspase-1–dependent maturation of IL-1β and IL-18, further amplifying neuroinflammation and contributing to microglial pyroptosis and neuronal injury. Unlike MAPK–NF-κB signaling, which initiates broad cytokine transcription, inflammasome activation sustains inflammation and exacerbates tissue damage during prolonged or repeated 1,2-DCE exposure. Experimental studies also demonstrate that NLRP3 activation is accompanied by engagement of caspase-3–mediated apoptotic pathways following 1,2-DCE exposure, which contributes to neuronal apoptosis and further exacerbates neuroinflammatory injury (31). In the same experimental context, mitochondria-dependent apoptotic signaling has been implicated in 1,2-DCE–induced cortical injury, with microRNA-mediated regulation of phospholipase D1 further linking mitochondrial dysfunction to neuronal apoptosis (31). Yin et al. reported that 28-day repeated inhalation of 1,2-DCE caused cognitive impairment, increased brain water content, and elevated levels of IL-1β, IL-6, and TNF-*α*, alongside upregulation of NLRP3, ASC, and cleaved caspase-1 in the brain. Inhibition of heat shock protein 90 (Hsp90) with 17-AAG effectively suppressed inflammasome activation, reduced apoptosis, and alleviated cerebral inflammation *in vivo* and in BV2 cells. This study identified the Hsp90–NLRP3 axis as a potential therapeutic target for blocking inflammasome-mediated neuroinflammation and BBB injury (32).

### 3.3 Fractalkine (FKN)/CX3CR1 signaling and microglial polarization

Fractalkine (FKN)/CX3CL1 is a chemokine primarily expressed by neurons and endothelial cells, while its receptor CX3CR1 is located on microglia. The FKN/CX3CR1 axis modulates microglial activation states and serves as a regulatory checkpoint in neuroimmune communication. Disruption of this signaling during 1,2-DCE intoxication skews microglia toward a pro-inflammatory phenotype, enhancing cytokine production and reinforcing MAPK–NF-κB signaling. This pathway functions primarily as a modulatory mechanism, shaping the magnitude and duration of inflammatory responses rather than initiating them. Beyond canonical cytokine signaling, neuron–microglia communication pathways such as the fractalkine (FKN)/CX3CR1 axis have been shown to exert stage-dependent effects on neuroinflammation in 1,2-DCE intoxication models. Recent studies demonstrate that early potentiation of FKN signaling may attenuate microglial pro-inflammatory polarization, whereas dysregulated or excessive activation at later stages can exacerbate neuroinflammation, highlighting the temporal complexity of glial regulatory mechanisms (33). Yang et al. demonstrated that early exposure to 1,2-DCE transiently enhanced FKN signaling, but prolonged intoxication markedly suppressed CX3CR1 expression, resulting in microglial hyperactivation and increased TNF-*α*, IL-6, and iNOS levels. Overexpression of CX3CR1 or administration of low-dose soluble FKN (sFKN) restored anti-inflammatory polarization of microglia and astrocytes and attenuated MAPK–NF-κB activation. However, excessive FKN signaling had the opposite effect, indicating a dose-dependent dual role of this pathway in neuroinflammation (33).

### 3.4 ERBB4/REPS2 signaling and astrocyte-driven inflammatory propagation

At later stages of intoxication, astrocyte-driven mechanisms become increasingly important, with ERBB4/REPS2 signaling contributing to the propagation and spatial spread of neuroinflammation. Overactivation of the ERBB4/REPS2 pathway promotes A1-type reactive astrocyte formation and excessive release of inflammatory mediators, further impairing BBB integrity. This astrocyte-centered pathway contributes to spatial propagation of inflammation across the neurovascular unit.

Du et al. (2024) found that 1,2-DCE exposure upregulated GFAP, C3, IL-1β, and TNF-*α* expression in astrocytes through overactivation of the ERBB4/REPS2 pathway, leading to inflammatory injury of the BBB. Treatment with human umbilical cord mesenchymal stem cells (HUCMSCs) or their miR-3064-5p–enriched exosomes markedly suppressed ERBB4 signaling, reduced cytokine release, and improved BBB integrity. These findings emphasize the potential of cell-free exosome therapy in mitigating astrocyte-driven neuroinflammation (18).

### 3.5 2-Chloroethanol–induced glial crosstalk as an upstream trigger

At the upstream metabolic level, the primary metabolite 2-chloroethanol (2-CE) acts as a critical trigger linking 1,2-DCE metabolism to subsequent glial activation and inflammatory amplification. By inducing reactive astrocytes and promoting astrocyte–microglia coupling, 2-CE reinforces MAPK–NF-κB signaling and downstream inflammasome activation, effectively linking metabolic toxicity to sustained neuroinflammatory injury.

Wang et al. demonstrated that 2-CE exposure activated p38 MAPK, AP-1, and NF-κB pathways, leading to RA formation and pro-inflammatory cytokine release. Pretreatment with fluorocitrate (FC), GIBH-130 (GI), or diacerein (Dia) inhibited RA formation and attenuated signaling activation. Notably, GI and Dia restored anti-inflammatory microglial polarization, while FC selectively blocked pro-inflammatory activity. These results provide mechanistic evidence that 2-CE–induced astrocyte–microglia coupling via MAPK–NF-κB pathways plays a pivotal role in 1,2-DCE-induced brain edema pathogenesis (34).

**Figure 1:**
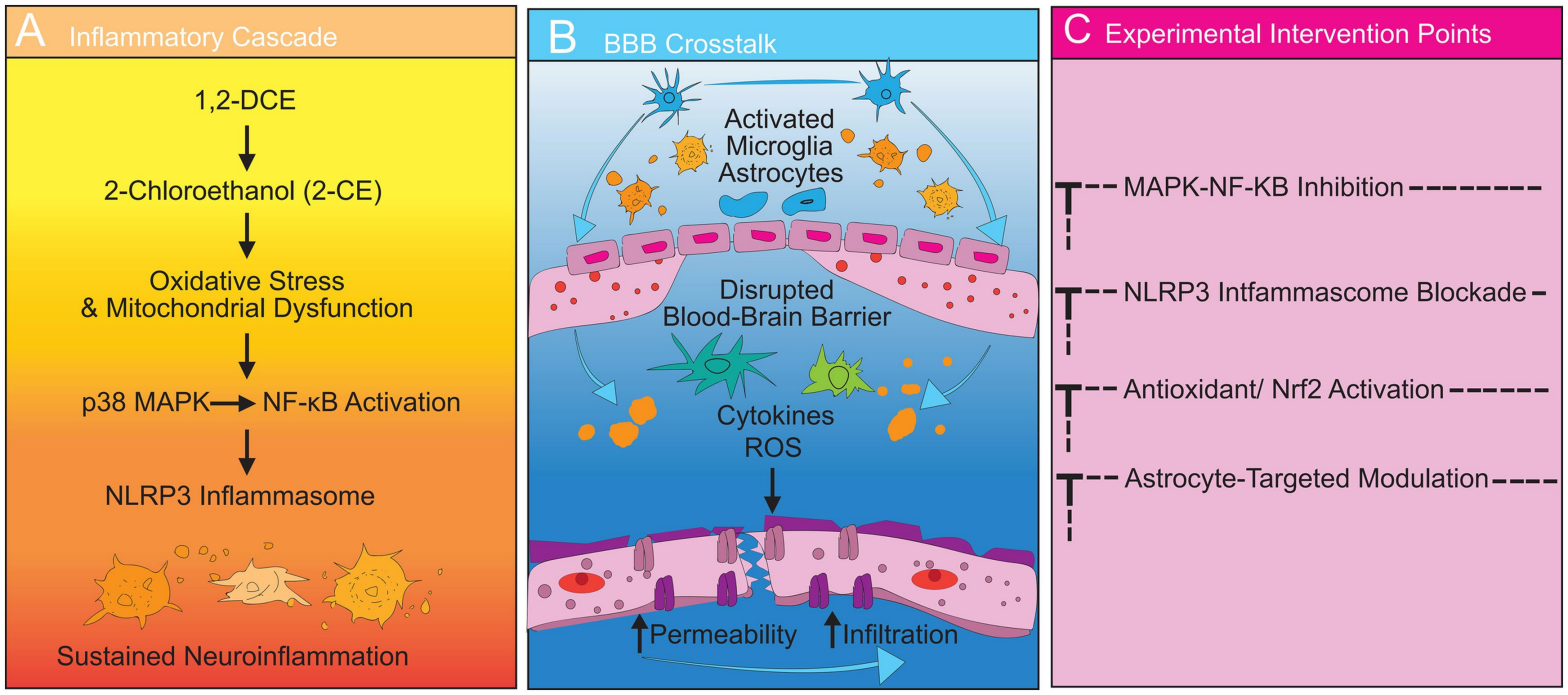
Integrated multi-panel schematic of inflammatory cascades, inflammation–BBB crosstalk, and experimental intervention points in 1,2-DCE-induced brain edema. **(A)** Hierarchical inflammatory cascade triggered by 1,2-DCE exposure. Metabolic conversion to 2-CE induces oxidative stress and mitochondrial dysfunction, leading to activation of p38 MAPK and subsequent NF-κB–dependent transcription of pro-inflammatory mediators. Persistent signaling further engages secondary inflammatory mechanisms, including NLRP3 inflammasome activation and sustained cytokine release. **(B)** Bidirectional crosstalk between neuroinflammation and BBB disruption. Pro-inflammatory cytokines and reactive oxygen species released by activated microglia and astrocytes promote tight-junction degradation and increased BBB permeability. BBB breakdown, in turn, facilitates the extravasation of blood-derived factors and immune cells, amplifying neuroinflammatory responses and establishing a self-sustaining positive feedback loop. **(C)** Experimental intervention points mapped onto the inflammatory cascade and BBB dysfunction. Dashed inhibitory lines indicate preclinical, proof-of-concept targets, including modulation of MAPK–NF-κB signaling, NLRP3 inflammasome activation, oxidative stress pathways, and astrocyte-mediated inflammatory propagation. These intervention points represent mechanistic vulnerabilities identified in experimental models rather than established clinical treatments.

In summary, these findings indicate that multiple signaling axes—including MAPK–NF-κB, NLRP3 inflammasome, FKN/CX3CR1, ERBB4/REPS2, and 2-CE–mediated astrocyte–microglia coupling—form an interconnected inflammatory network. Activation of these cascades leads to cytokine release, MMP-9–driven extracellular matrix degradation, and loss of tight-junction integrity, resulting in increased BBB permeability and brain edema (Table 1). Based on the above inflammatory-mediated cellular and molecular events, the overall mechanism is illustrated in Figure 1, which illustrates the key inflammatory chain from 1,2-DCE exposure to cerebral edema formation (Figure 1).

**Table 1:** Key inflammatory mediators and pathways in 1,2-DCE–induced brain edema.

Signal pathway	Primary activation trigger	Major cytokines /effectors	Principal cell types	Pathological consequence	Reference
p38 MAPK–NF-κB	Oxidative stress, ROS, Ca^2+^ overload	IL-1β, TNF-*α*, ICAM-1, VCAM-1, iNOS, MMP-9	Microglia, Astrocytes, Endothelium	Tight-junction degradation, BBB leakage	(16, 17)
NLRP3 inflammasome / Hsp90 Axis	Mitochondrial ROS, ATP depletion	IL-1β, IL-6, TNF-α, Caspase-1 activation	Microglia	Apoptosis, pyroptosis, BBB inflammation	(32)
FKN / CX3CR1 signaling	Loss of neuronal–microglial communication	TNF-α, IL-6, iNOS	Microglia, Astrocytes	Dysregulated polarization, chronic inflammation	(33)
ERBB4 / REPS2 pathway	Astrocytic overactivation by 1,2-DCE metabolites	IL-1β, TNF-α, C3, GFAP	Astrocytes	Astrocyte-mediated BBB injury	(18)
2-CE–induced MAPK–NF-κB / AP-1	2-chloroethanol accumulation	IL-1β, TNF-α, IL-6	Astrocytes → Microglia	RA formation, M1 polarization, BBB disruption	(34)

## 4 Crosstalk between inflammation and BBB disruption

The bidirectional crosstalk between inflammation and BBB disruption has been firmly established in diverse models of neurotoxicity and brain injury. During exposure to 1,2-DCE, cerebral hemorrhage, or metabolic disorders, inflammatory responses and structural BBB damage amplify one another, leading to a self-perpetuating cycle of neurovascular dysfunction. The BBB forms an integral component of the neurovascular unit, consisting of endothelial cells, pericytes, astrocytes, and neurons, and is primarily maintained by tight junction (TJ) proteins such as occludin, claudin-5, and ZO-1, which are essential for vascular integrity and selective permeability (35). Under inflammatory or oxidative stress conditions, activated microglia and astrocytes release abundant proinflammatory cytokines (TNF-*α*, IL-1β, IL-6) and ROS, which activate transcription factors including nuclear factor-κB (NF-κB), activator protein-1 (AP-1), and mitogen-activated protein kinase (MAPK) cascades. These signaling events promote the upregulation of matrix metalloproteinases (MMPs), particularly MMP-9, resulting in proteolytic degradation of TJ complexes, enhanced vascular permeability, and the development of vasogenic brain edema (16).

This mechanism is conserved across pathological conditions such as diabetes, hemorrhagic injury, and solvent-induced encephalopathy, where excessive MMP-9 and MMP-2 activity directly cleaves occludin and ZO-1, disrupting endothelial junctions and causing plasma extravasation (12, 35). Zlokovic proposed the “neurovascular unit inflammation model,” which posits that BBB dysfunction is not merely a downstream consequence of inflammation but also a principal driver of its propagation. Vascular rupture or xenobiotic-induced injury allows blood-derived molecules—such as fibrinogen, hemoglobin, and iron ions—to leak into the brain parenchyma, triggering microglial activation, pericyte loss, and a sustained cycle of neuroinflammation and oxidative stress. This process is accompanied by cytoskeletal reorganization in endothelial cells, upregulation of adhesion molecules (ICAM-1, VCAM-1), and infiltration of peripheral leukocytes, all of which further exacerbate BBB compromise (12, 13).

In the 1,2-DCE intoxication model, continuous phosphorylation of p38 MAPK enhances NF-κB and AP-1 activation, leading to robust MMP-9 expression and marked downregulation of TJ proteins (ZO-1, occludin, claudin-5). Pharmacological inhibition of p38 reverses these alterations, significantly decreasing brain water content and vascular leakage, indicating that inflammatory signaling directly contributes to BBB disruption and brain edema (17). Similarly, in hemorrhagic models, hemoglobin and its degradation products aggravate oxidative stress and inflammatory mediator release, further stimulating NF-κB, peroxisome proliferator–activated receptor gamma (PPARγ), and nuclear factor erythroid 2–related factor 2 (Nrf2) signaling (36). NF-κB activation sustains IL-1β, TNF-*α*, and MMP-9 expression, amplifying BBB injury, whereas PPARγ activation exerts a protective role by upregulating antioxidant enzymes (catalase, Cu/Zn–superoxide dismutase) and transcriptionally repressing NF-κB activity. The Nrf2 pathway complements this protection by inducing heme oxygenase-1 (HO-1) and glutathione-dependent detoxification systems, mitigating blood-derived oxidative toxicity.

Collectively, these findings reveal a unified mechanism of “inflammation–BBB” crosstalk: exogenous or endogenous insults initiate oxidative and inflammatory cascades, which activate MMPs to degrade TJ proteins, resulting in increased barrier permeability that facilitates the extravasation of inflammatory mediators and immune cells—thereby establishing a positive feedback loop. Targeting this loop through inhibition of MAPK–NF-κB signaling, suppression of MMP-9 activity, activation of Nrf2–PPARγ anti-inflammatory pathways, and preservation of endothelial energy homeostasis has shown significant potential in restoring BBB function and alleviating cerebral edema across experimental models (12, 13, 17, 35, 36).

## 5 Translational perspectives and experimental therapeutic targets

Current therapeutic strategies for 1,2-DCE-induced brain edema remain largely supportive, and no targeted treatments have been clinically validated. Mechanistic studies have nevertheless identified several molecular pathways that may represent experimental therapeutic targets. It is important to emphasize that most interventions discussed below are preclinical proof-of-concept tools rather than clinically applicable therapies.

### 5.1 Inhibition of MAPK and NF-κB signaling

The NF-κB and p38 MAPK pathways are central regulators of inflammatory cytokine release and MMP-9 activation during 1,2-DCE exposure. Pharmacological inhibitors such as BAY 11–7,082 and SB203580 have been shown to reduce microglial activation, lower TNF-*α* and IL-1β levels, and preserve tight-junction proteins (ZO-1, claudin-5) in experimental models (17, 18). These agents demonstrate that attenuation of early inflammatory signaling can reduce cytokine production, MMP-9 expression, and BBB permeability. However, their current utility is primarily mechanistic, and their clinical applicability remains limited due to off-target effects and safety concerns.

### 5.2 Targeting the NLRP3 inflammasome

Activation of the NLRP3 inflammasome drives the maturation of IL-1β and IL-18 and amplifies neuroinflammation. Yin et al. reported that inhibition of Hsp90 with 17-AAG blocked NLRP3 assembly and caspase-1 activation, alleviating cerebral inflammation and cognitive decline (32). These results suggest the Hsp90–NLRP3 axis as a novel therapeutic target. Selective inflammasome inhibitors (e.g., widely used experimental NLRP3 blockers) could complement anti-inflammatory approaches and preserve BBB integrity.

### 5.3 Enhancing endogenous antioxidant defense pathways

Excessive ROS and mitochondrial dysfunction play crucial roles in 1,2-DCE-induced oxidative injury. Enhancing the Nrf2–HO-1 antioxidant pathway upregulates SOD, CAT, and glutathione-related enzymes, restoring redox balance and mitochondrial integrity (21, 22). Agents such as sulforaphane and dimethyl fumarate activate Nrf2 signaling and suppress lipid peroxidation, while PPARγ agonists complement this effect by transcriptionally repressing NF-κB. Co-activation of Nrf2 and PPARγ may therefore provide a conceptual framework for further experimental investigation into oxidative–inflammatory interactions. Rather than targeting individual inflammatory mediators, modulation of redox homeostasis may attenuate multiple upstream triggers of inflammation and BBB injury. This approach warrants further investigation under exposure-relevant conditions.

### 5.4 Stem-cell-derived exosomes and BBB repair

Beyond pharmacological inhibition, Stem-cell–derived exosomes have shown promise in experimental models by modulating astrocytic signaling and preserving BBB integrity. Exosomes derived from human umbilical cord mesenchymal stem cells (HUCMSCs) containing miR-3064-5p were found to suppress ERBB4 signaling in astrocytes, reduce IL-1β and TNF-*α* expression, and preserve BBB structure (19). Such cell-free biotherapies may deliver anti-inflammatory microRNAs across the BBB and promote endogenous repair. Nevertheless, challenges related to scalability, delivery, dosing, and long-term safety currently limit their clinical feasibility. At present, such approaches should be viewed as emerging regenerative concepts rather than established therapeutic modalities. In summary, targeting MAPK–NF-κB, NLRP3, and Nrf2/PPARγ pathways—together with regenerative exosome-based therapies—offers an integrated framework to attenuate inflammation, oxidative stress, and vascular injury in 1,2-DCE-induced brain edema.

## 6 Concluding remarks and future perspectives

It should be noted that the inhalation concentrations used in experimental models often exceed current occupational exposure limits, and are primarily designed to elicit reproducible mechanistic endpoints rather than to replicate real-world exposure scenarios. 1,2-DCE–induced brain edema represents a complex and multifactorial neurotoxic condition arising from systemic chemical exposure. Clinically, it manifests as acute neurological deterioration—ranging from dizziness and headache to cognitive decline and coma—often without specific structural abnormalities at the early stage of imaging. Mechanistically, the condition reflects a convergence of oxidative stress, inflammation, and vascular dysfunction within the central nervous system (CNS). In this review, we comprehensively summarized emerging molecular and cellular pathways underlying 1,2-DCE-related brain edema, emphasizing BBB disruption, glial activation, oxidative stress–driven inflammation, mitochondrial failure, and the bidirectional feedback between vascular and neuroinflammatory injury.

Mounting evidence positions BBB dysfunction as both an initiating and amplifying event in 1,2-DCE neurotoxicity. Damage to endothelial cells and downregulation of tight-junction proteins (ZO-1, occludin, claudin-5) allow circulating cytokines, toxic metabolites, and immune cells to penetrate the CNS, setting in motion a cascade of glial activation and neuroinflammation. Within this permissive environment, microglia and astrocytes transition into reactive states, releasing IL-1β, TNF-*α*, IL-6, and nitric oxide, thereby intensifying oxidative and nitrosative stress. Concurrent mitochondrial dysfunction further disrupts energy metabolism, depleting ATP and promoting calcium overload, which synergize to aggravate cytotoxic and vasogenic edema. The culmination of these processes compromises synaptic homeostasis and neuronal survival, potentially leading to irreversible cognitive impairment and long-term neurological sequelae.

An important conceptual advancement emerging from recent research is the recognition that 1,2-DCE neurotoxicity is not a uniform entity but rather a spectrum of overlapping pathophysiological patterns. Depending on exposure duration, metabolic status, and genetic background, individuals may exhibit oxidative-dominant, inflammatory-dominant, or vascular-permeability–dominant subtypes. This heterogeneity has significant implications for prognosis and therapeutic targeting. The integration of transcriptomic, proteomic, and metabolomic analyses is beginning to reveal distinct molecular phenotypes of 1,2-DCE-induced injury, each governed by different signaling axes such as CYP2E1-mediated oxidative metabolism, p38 MAPK–NF-κB-driven cytokine cascades, or NLRP3 inflammasome activation.

Despite these mechanistic insights, major translational and clinical gaps persist. Reliable non-invasive biomarkers for early detection of 1,2-DCE neurotoxicity and for monitoring the dynamics of BBB integrity are lacking. Current diagnosis still relies heavily on clinical presentation and neuroimaging, often at stages when injury is already advanced. Likewise, there are no approved pharmacological therapies that specifically target the oxidative, inflammatory, or endothelial components of 1,2-DCE-induced brain injury. Existing interventions remain largely supportive, focusing on exposure cessation, osmotic therapy, and general neuroprotection. This stagnation underscores an urgent need for mechanism-based diagnostic and therapeutic innovations.

Looking forward, future research should aim to construct an integrated molecular framework linking exposure dose, metabolic activation, and CNS outcomes. The development of biomarker panels combining serum metabolites (e.g., 2-chloroethanol derivatives), cerebrospinal fluid cytokine profiles, and advanced neuroimaging signatures (BBB permeability mapping, microstructural MRI) could revolutionize early diagnosis and risk stratification. Mechanistically driven interventions should focus on restoring BBB integrity, attenuating glial overactivation, and stabilizing mitochondrial function. Promising candidates include inhibitors of p38 MAPK, NF-κB, and MMP-9; antagonists of the NLRP3 inflammasome; and activators of the Nrf2/HO-1 or PPARγ pathways. Stem cell–derived exosomes enriched in anti-inflammatory microRNAs, such as those from human umbilical cord mesenchymal stem cells, may offer novel cell-free therapeutic strategies to suppress astrocytic ERBB signaling and promote BBB repair.

In addition, longitudinal cohort studies following occupationally exposed populations are urgently needed to define the natural course of cognitive recovery, emotional regulation, and long-term neurodegenerative risk after 1,2-DCE intoxication. Such data will be essential for developing preventive surveillance programs and rehabilitation strategies.

In summary, 1,2-DCE-induced brain edema exemplifies a paradigm of “chemical–neurovascular–immune” crosstalk, in which systemic xenobiotic exposure disrupts CNS homeostasis through interlocking oxidative and inflammatory pathways. Elucidating these mechanisms in finer temporal and spatial resolution will not only deepen our understanding of solvent-induced neurotoxicity but also lay the groundwork for targeted, disease-modifying interventions. The continued integration of mechanistic studies with translational and occupational health research is critical to transforming these molecular insights into tangible improvements in prevention, diagnosis, and neuroprotection for affected individuals.
